# Modelling Nonalcoholic Steatohepatitis In Vivo—A Close Transcriptomic Similarity Supports the Guinea Pig Disease Model

**DOI:** 10.3390/biomedicines9091198

**Published:** 2021-09-10

**Authors:** Josephine Skat-Rørdam, David H. Ipsen, Stefan E. Seemann, Markus Latta, Jens Lykkesfeldt, Pernille Tveden-Nyborg

**Affiliations:** 1 Section of Experimental Animal Models, Department of Veterinary and Animal Sciences, Faculty of Health and Medical Sciences, University of Copenhagen, DK-1870 Frederikberg, Denmark; jsr@sund.ku.dk (J.S.-R.); dhi@sund.ku.dk (D.H.I.); jopl@sund.ku.dk (J.L.); 2 Center for non-coding RNA in Technology and Health, Department of Veterinary and Animal Sciences, Section for Animal Genetics, Bioinformatics and Breeding, University of Copenhagen, DK-1871 Frederiksberg, Denmark; seemann@rth.dk; 3 Liver Disease Research, Global Drug Discovery, Novo Nordisk A/S, DK-2760 Måløv, Denmark; mrlq@novonordisk.com

**Keywords:** nonalcoholic steatohepatitis, fibrosis, transcriptome, animal model, guinea pig, biomarkers

## Abstract

The successful development of effective treatments against nonalcoholic steatohepatitis (NASH) is significantly set back by the limited availability of predictive preclinical models, thereby delaying and reducing patient recovery. Uniquely, the guinea pig NASH model develops hepatic histopathology and fibrosis resembling that of human patients, supported by similarities in selected cellular pathways. The high-throughput sequencing of guinea pig livers with fibrotic NASH (*n* = 6) and matched controls (*n* = 6) showed a clear separation of the transcriptomic profile between NASH and control animals. A comparison to NASH patients with mild disease (GSE126848) revealed a 45.2% overlap in differentially expressed genes, while pathway analysis showed a 34% match between the top 50 enriched pathways in patients with advanced NASH (GSE49541) and guinea pigs. Gene set enrichment analysis highlighted the similarity to human patients (GSE49541), also when compared to three murine models (GSE52748, GSE38141, GSE67680), and leading edge genes *THRSP, CCL20* and *CD44* were highly expressed in both guinea pigs and NASH patients. Nine candidate genes were identified as highly correlated with hepatic fibrosis (correlation coefficient > 0.8), and showed a similar expression pattern in NASH patients. Of these, two candidate genes (*VWF* and *SERPINB9*) encode secreted factors, warranting further investigations as potential biomarkers of human NASH progression. This study demonstrates key similarities in guinea pig and human NASH, supporting increased predictability when translating research findings to human patients.

## 1. Introduction

Hepatic fibrosis is the primary prognostic marker of mortality in nonalcoholic steatohepatitis (NASH) globally affecting millions [1]. However, treatment options are scarce, and drug development is hampered by the lack of animal models reflecting the disease spectrum and etiology. Though frequently applied, many mouse and rat models do not develop NASH with advanced fibrosis (bridging fibrosis and cirrhosis) without the use of hepatotoxins and micronutrient-deficient diets, consequently compromising construct and, by extension, predictive validity [2,3,4].

Uniquely, guinea pigs develop NASH with advanced fibrosis when subjected to a westernised diet [5,6,7]. Similar to human NASH, lesions originate from the hepatic central veins and sequentially progress from mild fibrosis to advanced (bridging) fibrosis within 25 weeks [6,7]. Furthermore, guinea pigs and humans share an LDL-dominant lipoprotein profile in contrast to the HDL-dominant profile of rats and mice, and targeted analysis of genes related to hepatic lipid metabolism, inflammation, and fibrogenesis further supports a high degree of similarity between the guinea pig disease model and human NASH [8,9]. However, unbiased systematic analysis of the guinea pig NASH transcriptome has not previously been undertaken, and is impeded by the incompletely annotated guinea pig genome. Using RNA sequencing and human orthologue mapping to improve annotation, this paper investigates the translational validity of the guinea pig NASH transcriptome and directly compares transcriptome remodelling to profiles from two patient datasets representing either mild or advanced disease, and three frequently used mouse models. Lastly, we propose a selection of candidate genes present in both human and guinea pig fibrotic NASH that may prove valuable in future drug discovery and biomarker development.

## 2. Materials and Methods

### 2.1. Animals

Animal experiments were approved by the Animal Experimentation Inspectorate under the Danish Ministry of the Environment and Food, and in accordance with the European Legislation of Animal Experimentation 2010/63/EU.

This study utilises a subset of liver samples, collected from intact nonperfused livers, from a previously published study [6]. Briefly, 10-week-old female Hartley guinea pigs (Charles River Laboratory, Lyon, France) were allowed one week of acclimatisation before being block-randomised into groups on the basis of body weight. Consistent with previous studies, this study was only performed on female guinea pigs, as hierarchical fighting in males is a critical welfare concern in long-term studies of this kind. The guinea pigs were fed a high-fat (20% fat, 15% sucrose, and 0.35% cholesterol) or chow (4% fat, 0% sucrose, 0% cholesterol) diet for 25 weeks. The exact dietary compositions were published elsewhere [6]. Six high-fat-fed animals with grade 3 (bridging) fibrosis and six randomly selected control animals without NASH and fibrosis were used for RNA sequencing.

At termination, the animals were preanaesthetised with 0.8 mL/kg body weight Zoletil mix (125 mg tiletamine (0.93 mg/kg), 125 mg zolazepam (0.93 mg/kg, Zoletil 50 Virbac Laboratories, Carros, France) + 200 mg xylazine (1.49 mg/kg, Narcoxyl vet 20 mg/mL; Intervet International, Boxmeer, Holland) + 7.5 mg butorphanol (0.06 mg/kg; Torbugesic vet 10 mg/mL; Scanvet, Fredensborg, Denmark) diluted 1:10 in isotonic NaCl), placed on isoflurane (3–5%) and, following the disappearance of intradigital reflexes, euthanised by decapitation as previously described [6].

### 2.2. Liver Samples and Histology

Liver samples were collected from the left lateral lobe (lobus hepatis sinister lateralis), snap-frozen in liquid nitrogen, stored at −80 ^°^C or fixed in paraformaldehyde, and subsequently embedded in paraffin and stained with haematoxylin and eosin or Masson’s trichrome for histological evaluation as previously published [6]. Liver histology was scored in accordance with guidelines by Kleiner et al. [10]. Steatosis was scored as 0 (5%), 1 (5–33%), 2 (>33–66%), or 3 (>66%) of the overall parenchymal tissue. Ballooning was scored as 0 (none), 1 (few/minimal), or 2 (many/prominent). Inflammation was scored in three lobuli, defined by two portal areas and one central vein as 0 (no foci), 1 (1 foci), 2 (2–4 foci), or 3 (>4 foci) per × 200 field. A focus was defined as ≥3 inflammatory cells in close proximity. Fibrosis was scored as 0 (none), 1A (mild, zone 3 perisinusoidal), 1B (moderate, zone 3 perisinusoidal), 1C (periportal), 2 (perisinusoidal and periportal), 3 (bridging), or 4 (cirrhosis). All scorings were performed in a randomised and blinded manner, and were previously published [6]. For the quantification of the relative fibrosis area, sections were stained with Picro Sirius Red and Weigert’s haematoxylin. Total collagen area was analysed on digital images of the entire liver section using VisioPharm Image analysis (version 2020.01.3.7887, VisioPharm, Hørsholm, Denmark) in accordance with quantifications of picrosirius red staining in liver samples from preclinical models and human patients [11,12]. Fibrosis area is shown in Supplementary Table S1, Additional File S1.

### 2.3. Guinea Pig Hepatic RNA Sequencing

RNA sequencing was performed on 12–24 mg of liver tissue from control (*n* = 6) and NASH animals with bridging fibrosis (*n* = 6). RNA extraction, purification, and sequencing were performed by QIAGEN Genomic Services (QIAGEN, Hilden, Germany). Briefly, library preparation was performed using a TruSeq^®^ Stranded mRNA Sample preparation kit (Illumina Inc., San Diego, CA, USA), and library size distribution was validated and quality-inspected on a Bioanalyzer 2100 (Agilent Technologies, Santa Clara, CA, USA). Subsequently, single-read 75-nucleotide read-length sequencing was performed on the Illumina NextSeq 500 platform with 30 million reads per sample according to the manufacturer’s specifications (Illumina Inc., San Diego, CA, USA). Sequencing data are available at GEO expression omnibus with accession number GSE158168.

### 2.4. Transcriptome Analyses

FASTQ files obtained from QIAGEN Genomic Services were processed in Trimmomatic (version 0.38.0) [13]. Reads shorter than 50 bases were removed, and all reads were trimmed of the leading 9 bases due to base-call quality below a Phred score of 32 and biased sequence composition. Trimmed reads were mapped to the Ensembl Cavia Porcellus genome Cav.Por.3.0 (Ensembl release 98, September 2019) with Hisat2 (version 2.1.0); multimapped reads were ignored for further analysis [14]. Subsequent transcript assembly and quantification were performed using Stringtie and Stringtie merge (version 1.3.6) [15], using the Cav Por.3.0 gene annotation as a guide. As the guinea pig genome is incompletely annotated, BioMart (version 2.42.0) [16] was used instead to obtain human orthologues (GRCh38.p13, Ensembl release 98, September 2019). Although the evolutionary distance of the guinea pig genome is marginally smaller to that of mice than to that of humans [17], many of the regulatory mechanisms of the immune system are more comparable between humans and guinea pigs [18]. Human orthologues were obtained with a sequence identity cut-off >50% using the Bioconductor package (version 3.10) [19]. Genes without annotation were excluded from analysis, resulting in a total of 17,332 unique genes. To diminish background noise, genes with total read count < 200 across all samples were excluded from subsequent analysis, producing a final list of genes containing 11,896 genes, for which differential expression was calculated using DESeq2 (version 1.26.0) [20].

### 2.5. Protein–Protein Association Network

A protein–protein association network was built using Cytoscape (version 3.8.0) [21] of the 100 proteins with the strongest association to NASH, as annotated by the DISEASES database [22,23]. Protein–protein associations were based on different confidence channels (e.g., physical association from experimental data and functional associations from curated pathways, automatic text mining, and prediction methods) provided by the STRING database [24,25]. A STRING confidence score of at least 0.7 was applied. For gene identifiers *CBLC, CCN1, RACK1, PCDHGA6*, and *HCN2,* no exact match was found by Cytoscape, and they were thus excluded from analysis.

### 2.6. Translational Aspects of the Guinea Pig Model

To investigate the translatability and benchmark the guinea pig as a model of NASH, the transcriptome was compared to two human datasets and three murine datasets (Table 1), which were all publicly available through the GEO expression omnibus [26]. Specifically, the guinea pig transcriptome was compared to human dataset (GSE126848) Human NASH1 (HNASH1) consisting of 14 healthy patients and 16 patients diagnosed with NASH (steatosis–activity–fibrosis ≥ 2) [27,28]. To determine if a core set of NASH-associated advanced-fibrosis genes could be identified for NASH irrespective of species, the guinea pig dataset was compared to human gene-expression data (GSE49541) Human NASH 2 (HNASH2), consisting of 40 patients with mild (F0–F1) nonalcoholic fatty liver disease (NAFLD) and 32 patients with advanced NAFLD and F3–F4 fibrosis [29]. In addition, guinea pig expression data were compared to two preclinical mouse NAFLD models, Western Diet 1 (WD1) (GSE52748) [30], Western Diet 2 (WD2) (GSE38141) [31], and one preclinical mouse NASH model, DIAMOND (GSE67680) [32] (Table 1). The mouse models were selected on the basis of similarity in dietary content (high fat, cholesterol, and sucrose), the availability of data, and the inclusion of a relevant control group without NASH and fibrosis.

### 2.7. Correlation Analysis of Gene Expression and Fibrosis Quantification

Genes potentially linked to advanced fibrosis stages were identified by correlating the normalised rlog-transformed values [20] for each gene to the relative fibrosis area of each animal (Supplementary Table S1, Additional File S1). We selected genes with a Pearson correlation coefficient of ≥0.8 for both all animals (control and NASH) and NASH animals alone. Additionally, the direction (negative or positive) of the Pearson correlation coefficient had to be identical for both groups (NASH animals alone and all animals). Lastly, only genes with log2 fold change >1 in the guinea pig dataset and a similar expression pattern (up- or downregulated) in the human dataset (GSE49541) were selected. For the regression analysis of each gene, see Additional File S2.

### 2.8. Statistical Analysis

Guinea pig and human dataset HNASH1 (GSE126848) were analysed by high-throughput sequencing [28]. Raw counts from RNA sequencing analysis were used as input for differential expression analysis performed with the DEseq2 package [20]. In contrast, human dataset HNASH2 (GSE49541) [29] and murine datasets (WD1 [30], WD2 [31], DIAMOND [32]) are array datasets, and the LIMMA package (version 3.42.2) [33] was used instead to identify differentially expressed genes (DEGs). For all datasets, DEGs were defined as genes with a Benjamini–Hochberg corrected *p*-value (*q*-value) < 0.05. For selection of the top 20 or top 200 DEGs in any dataset, the criterion for inclusion was *q* < 0.05, and genes were sorted by absolute log2 fold change. Gene set enrichment analysis (GSEA) was performed on log2 fold change pre-ranked values using the fgsea (version 1.12.0) package in R [34]. Pathways used as input were obtained from the Molecular Signature Database (MSigDB) [35,36,37]. Hallmark pathways [37] provided an overview when comparing animal and human datasets. For the analysis of a selected set of pathways, the Reactome pathways were applied [38]. When identifying the top 50 enriched Reactome pathways, the top 25 downregulated and the top 25 downregulated pathways were selected on the basis of their absolute normalised enrichment score and *q* < 0.1. The full analysis of Hallmark gene sets and leading edge genes defined as the genes accounting for most of the enrichment signal are shown in Supplementary Table S3, Additional File S1 [36]. Principal-component analysis was performed on normalised transformed values that had been obtained using Deseq2 [20] (GP, HNASH1) or LIMMA [33] (DIAMOND, WD1, WD2) packages. All genes were included unless otherwise stated. For correlation analysis, Pearson’s correlation was used and *p* values < 0.05 were considered statistically significant.

## 3. Results

### 3.1. Guinea Pig NASH Development and Disease Stage

**Figure 1:**
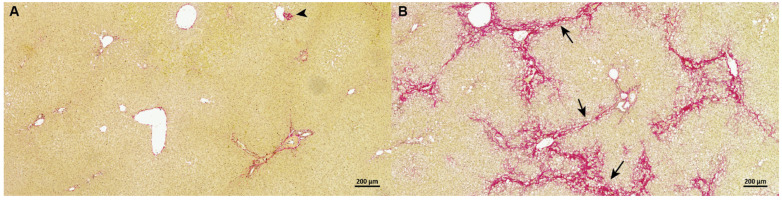
Representative pictures of the distribution of hepatic fibrotic tissue in control and NASH (stage F3) guinea pigs in Picro Sirius red stained sections. (**A**) In control animals, fibrous tissue (red) surrounds the central veins and portal areas, but does not expand into the hepatic parenchyma. An isolated small area of fibroplasia (arrowhead) can be seen as a normal occasional finding. (**B**) Bridging fibrosis (arrows) (F3 grade) is clearly evident in animals with NASH after 25 weeks on a high-fat diet.

High-fat-fed guinea pigs developed NASH with advanced bridging fibrosis (F3) as previously reported (Figure 1) [6]. Steatosis (*p* < 0.01), inflammation (*p* < 0.01), hepatocyte ballooning (*p* < 0.05), and fibrosis (*p* < 0.01) were evident in all NASH animals compared to healthy controls (Table 2) as previously reported [6]. In accordance with advanced fibrosis (F3) stage, the relative fibrosis area was significantly increased in NASH animals compared to in the controls (*p* < 0.001).

### 3.2. The Hepatic Transcriptome Clearly Distinguishes Guinea Pigs with NASH from Controls

**Figure 2:**
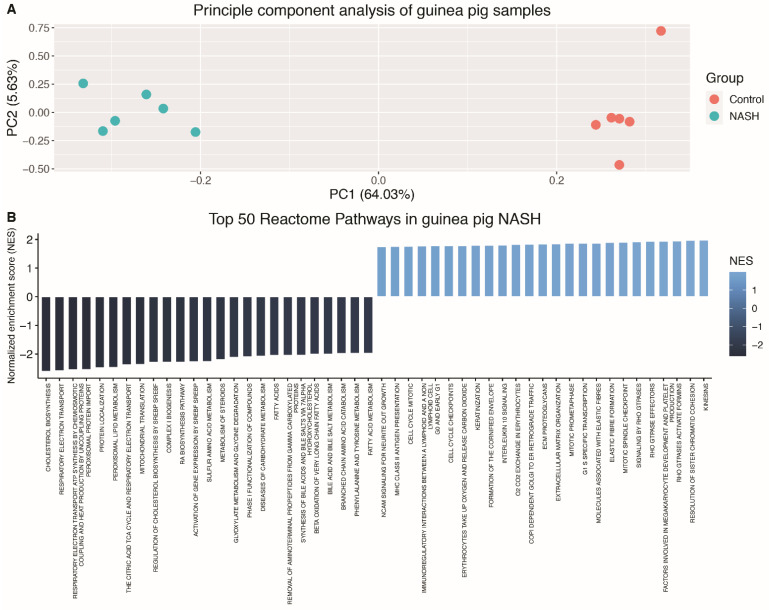
Principal-component analysis and top 50 dysregulated pathways in guinea pigs with NASH (**A**) Principal-component analysis plot of guinea pig samples. NASH and control animals clearly separated on the basis of all genes by PC1 explaining 64.03% of the total data variance. (**B**) Top 50 reactome pathways obtained from GSEA in guinea pigs with NASH vs. healthy controls. Top 50 pathways included the 25 most upregulated and the 25 most downregulated pathways. All pathways were selected on the basis of corrected Benjamini–Hochberg *p*-values and normalised enrichment score. PC: principal component, NASH: non-alcoholic steatohepatitis, NES: normalised enrichment score.

A total of 6683 DEGs were identified between guinea pigs with NASH and controls (*q* < 0.05). Principal-component analysis (Figure 2A) showed a clear separation of the two groups, corroborating distinct transcriptomic differences between NASH and control animals. The top 20 DEGs include genes involved in immune cell signalling (*ADAMDEC1*, *CCL7*, *TNFSF18*), cell-to-cell contact (*SPTA1*, *PAK6*, *DSG4*), cholesterol metabolism (*STAR*), and liver regeneration (*ECT2*) (Table 3). This is also reflected in the GSEA (Figure 2B), where the top 50 enriched pathways include extracellular-matrix organisation, MHC Class II antigen presentation, and cell-cycle mitosis. Among the downregulated pathways were cholesterol biosynthesis, bile acid and bile salt metabolism, the citric acid cycle and respiratory electron transport and fatty acid metabolism.

### 3.3. The Guinea Pig NASH Transcriptome Is Similar to That of Humans with Early-Stage NASH

**Figure 3:**
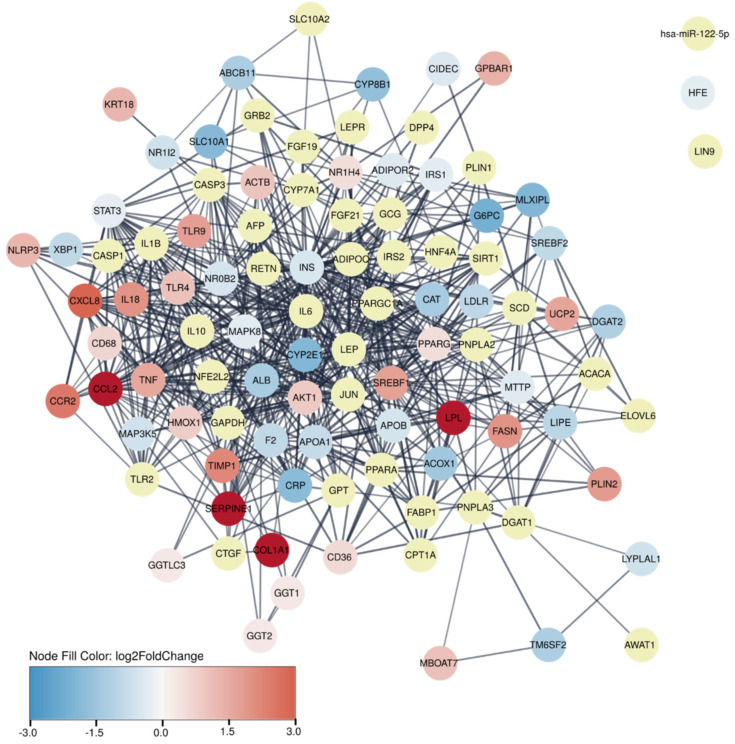
Protein–protein association network showing top 100 genes most strongly associated with human NASH. Yellow nodes indicate 40 proteins that are not differentially expressed in guinea pig NASH. Remaining network nodes are coloured by log2 fold changes of differentially expressed genes in guinea pigs using the default Cytoscape colour gradient blue–white–red of log2 fold change from −3 to 3.

**Figure 4:**
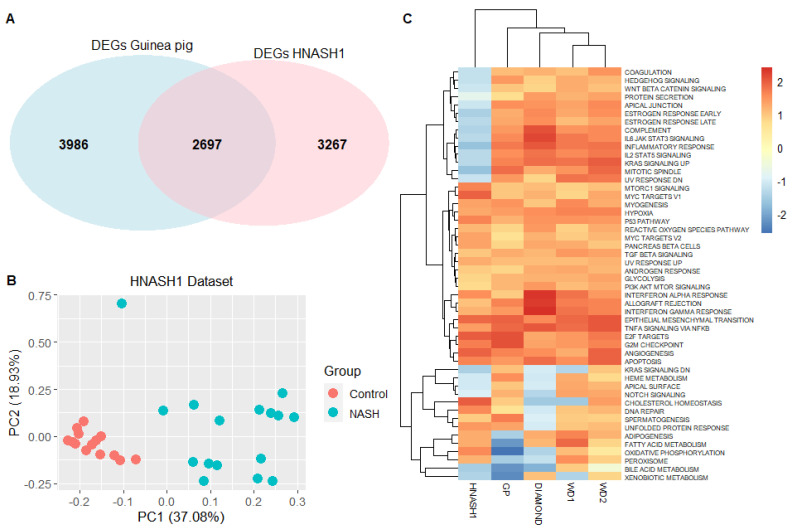
Comparison of preclinical models and patients with early-stage NASH. **(A)** Venn diagram of differentially expressed genes from HNASH1 (patients with mild disease vs. healthy controls) and guinea pig datasets, and their overlapping genes. Both datasets were analysed by DEseq2, and differentially expressed genes were selected on the basis of *q* < 0.05. (**B**) Principal-component analysis using top 200 differentially expressed genes in guinea pigs clearly separated patients with NASH from healthy controls in the HNASH1 dataset. Top 200 differentially expressed genes were selected on the basis of *q* < 0.05 and highest absolute log2 fold-change values. (**C**) Heatmap demonstrating overlap in expression patterns of Hallmark pathways. The heatmap is based on normalised enrichment scores from gene set enrichment analysis of Hallmark pathways from each dataset, i.e., HNASH1, guinea pig, and the included murine datasets (WD1, WD2, and DIAMOND). Dendrogram depicts hierarchical clustering of groups according to normalised enrichment scores. Colour bar indicates normalised enrichment scores, blue indicates a downregulated gene set, and red indicates an upregulated gene set. WD1 refers to GSE52748, WD2 refers to GSE38141, HNASH1 refers to GSE126848, DIAMOND refers to GSE67680. GP: guinea pig, DEG: differentially expressed gene, PC: principal component, NASH: nonalcoholic steatohepatitis.

The translational value of the guinea pig DEGs was assessed by comparing these to the 100 human genes most strongly associated with NASH, as annotated in the DISEASE database [22,23]. This comparison revealed that 60% of the human genes were differentially expressed in guinea pigs, whereas 40% were not, and is visualised as a protein–protein association network in Figure 3. To further substantiate the translational findings, RNA sequencing results were also compared to a human dataset with early-stage NASH patients and healthy controls (HNASH1, GSE126848) [28]. Similar to the number of DEGs identified in guinea pigs, a total of 5964 DEGs (q < 0.05) were identified in the human dataset, and of these, 2697 (45.2%) genes were identical to the DEGs in the guinea pigs (Figure 4A). Moreover, the top 200 DEGs from the guinea pig dataset were sufficient to distinguish patients with early-stage NASH from healthy controls by using principal-component analysis (Figure 4B).

To benchmark these results in relation to other preclinical models of NAFLD/NASH (WD1, WD2, DIAMOND), the similarity of the animal models to the human dataset (GSE126848) was compared by investigating the comparability of enriched pathways. Figure 4C shows a heatmap based on normalised enrichment scores of all 50 hallmark pathways in the different animal and human datasets (GSE126848). The three murine models group together more closely compared to the guinea pig, and all four rodent models are more similar to each other than to the human dataset. Importantly, the GSE126848 dataset is derived from patients with early NASH as opposed to the more severe NASH in the guinea pigs, which may reduce comparability between groups. In contrast to the animal models, 14 pathways were uniquely downregulated in the human dataset. These 14 pathways include inflammatory signalling (e.g., inflammatory response (*q* < 0.01), complement (*q* < 0.05), and IL2 STAT5 signalling (*q* < 0.1)). Leading edge genes specifically revealed *CD44* and *CCL20* (C–C motif chemokine ligand 20) to be differentially regulated in human and guinea pig datasets (Supplementary Table S3, Additional File S1). The downregulation of adipogenesis (*q* < 0.05) and fatty acid metabolism (*q* < 0.01) was only found in guinea pigs.

### 3.4. Guinea Pig NASH Transcriptome Resembles Human Advanced NASH

**Figure 5:**
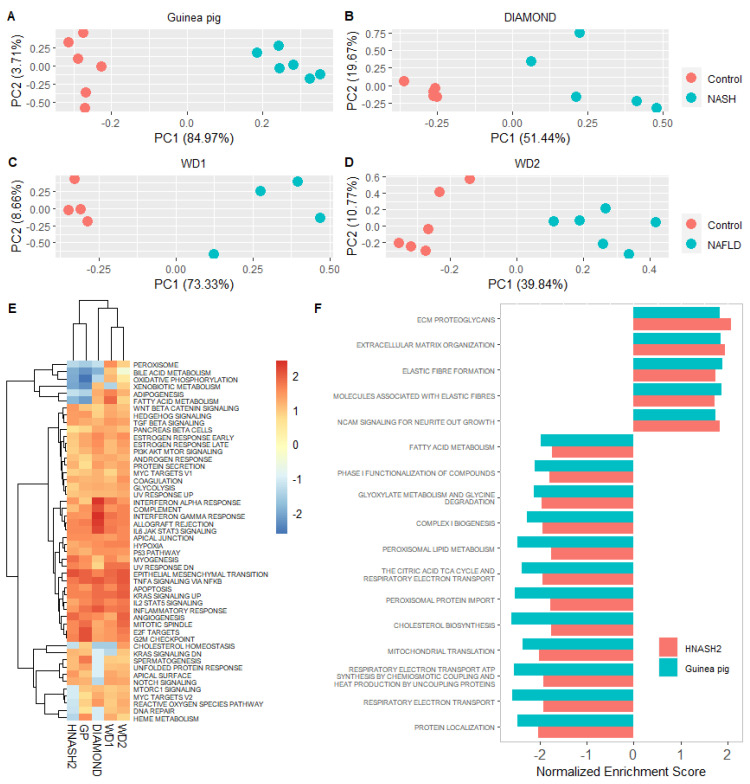
Comparison of preclinical models and patients with advanced NASH. (**A**–**D**) Principal-component analysis using top 200 differentially expressed genes from HNASH2 (NASH patients with advanced vs. mild fibrosis) could separate NASH/NAFLD from healthy controls in all included preclinical models. Differentially expressed genes from HNASH2 dataset selected on the basis of *q* < 0.05 and highest absolute log2 fold change. Principal-component analysis plots depict normalised and transformed values for the 200 genes in each of the animal datasets. (**E**) Heatmap demonstrating overlap in expression patterns of Hallmark pathways. Heatmap is based on normalised enrichment scores from the gene set enrichment analysis of Hallmark pathways from each dataset. Dendrogram depicts hierarchical clustering of groups according to normalised enrichment scores. Colour bar indicates normalised enrichment scores for each gene set. Blue indicates a downregulated gene set, whereas red indicates an upregulated gene set. (**F**) Overview of normalised enrichment scores of the 17 pathways in common between HNASH2 and guinea pigs. Top 50 pathways of the HNASH2 dataset were compared with the top 50 enriched pathways in guinea pigs. Top 50 pathways included the top 25 most upregulated and the top 25 most downregulated pathways. All pathways were selected on the basis of corrected Benjamini–Hochberg *p*-values and normalised enrichment scores. WD1 refers to GSE52748, WD2 refers to GSE38141, DIAMOND refers to GSE67680, HNASH2 refers to GSE49451. GP: guinea pig, PC: principal component, NAFLD: nonalcoholic fatty liver disease, NASH: nonalcoholic steatohepatitis.

The translatability of the guinea pig transcriptome was also assessed in relation to patients with advanced disease. In contrast to the HNASH1 dataset, where most patients had ≤1 grade fibrosis (1 of 16 patients had grade 2 fibrosis), and all patients had ≤1 grade inflammation, the HNASH2 dataset compares NAFLD patients with either mild (grade 0–1) or severe (grade 3–4) fibrosis [28,29]. The top 200 DEGs from these patients distinguished guinea pigs and WD1 with NAFLD/NASH from the controls (Figure 5A,C). DIAMOND and WD2 animals could also be separated by these DEGs (Figure 5B,D), but their fraction of variance explained by first principal component PC1 was lower than that of guinea pigs and WD1.

In the GSEA, murine (WD1, WD2 and DIAMOND) models clustered, while guinea pigs and the HNASH2 dataset formed a separate cluster (Figure 5E). To explore this relationship, the top 50 enriched Reactome pathways in the HNASH2 dataset were compared to the top 50 enriched Reactome pathways in each of the preclinical models. Guinea pig and HNASH2 datasets share 17/50 (34%) enriched pathways, whereas the WD2, WD1, DIAMOND mice and HNASH2 datasets share 17/50 (34%), 7/50 (14%), and 9/50 (18%), respectively (see Supplementary Table S2, Additional File S1 for a list of matching pathways). The 17 pathways shared between guinea pigs and the patients with advanced fibrosis were all regulated in the same direction with 12 downregulated and 5 upregulated pathways (Figure 5F). Consistent with advanced fibrosis in both guinea pigs and patients, the upregulated pathways (extracellular metabolism, ECM proteoglycans, elastic-fibre formation, molecules associated with elastic fibres) were linked to fibrosis. Downregulated pathways included mitochondrial processes such as the citric acid cycle and respiratory electron transport, peroxisomal protein import, mitochondrial translation, and fatty acid metabolism. Further evaluation of fatty acid metabolism leading edge genes revealed thyroid hormone responsive (*THRSP*) to be differentially regulated in HNASH2 and guinea pig datasets, whereas it was upregulated in HNASH1 (Supplementary Table S4, Additional File S1).

### 3.5. Identification of Potential New Biomarkers of Fibrosis Deposition

To identify genes directly related to the amount of fibrosis in our NASH guinea pigs, the relative fibrosis amount was correlated to the expression of the 11,896 identified genes. Only genes with a correlation coefficient of ≥0.8 and a log2 fold change of ≥1 were included. Each gene was compared to the two human datasets HNASH1 and HNASH2, which consisted of NASH patients with mild disease and NASH patients with advanced fibrosis (grade 3–4), respectively. The final list comprises nine genes: *ACKR3, BIRC3, CHST11, EMP3, FZD7, RGS14, RHBDF1, SERPINB9*, and *VWF* (Table 4; for regression analysis, see Additional File S2).

## 4. Discussion

This paper shows the first comparison of human and guinea pig NASH transcriptomes, and reveals the high translational potential of this model compared to the included murine models. DEGs clearly separated guinea pigs with NASH from healthy controls, and GSEA revealed an over-representation of fibrosis-related signalling, while energy-generating processes were downregulated. Importantly, guinea pigs with NASH and advanced fibrosis (F3) recapitulate the transcriptional profile of NASH patients with advanced (F3–F4) fibrosis, emphasising that the guinea pig NASH model possesses high translational potential, which can be used in drug and biomarker discovery.

A comparison of the guinea pig transcriptome to the human dataset (GSE126848 (HNASH1)) demonstrated that the top 200 DEGs in NASH guinea pigs were able to separate patients with NASH from healthy individuals [28]. This similarity was further substantiated by the 60% overlap in guinea pig NASH DEGs and human genes from the DISEASE database [23], and a separate grouping of guinea pigs and patients from the HNASH2 dataset in the GSEA compared to the murine models. However, some pathways displayed altered regulation in guinea pigs compared to the HNASH2 dataset, including heme metabolism, cholesterol homeostasis, and the reactive-oxygen-species pathway. In this aspect, differences in pathways associated with cholesterol homeostasis are not surprising, as the guinea pig HF diet contains an excess (0.35%) of cholesterol. Differences in the content and composition of dietary fatty acids may also induce alterations in hepatic metabolism and associated pathways that may influence end points and limit comparisons between studies, underlining controlled dietary regimes as a central point of attention when modelling this disease [47]. Similar to humans with advanced NASH (HNASH2), adipogenesis and fatty acid metabolism gene sets were downregulated in the guinea pig, in contrast to humans with mild disease (HNASH1) and the murine datasets (WD1, WD2 and DIAMOND). Fatty acid metabolism was also among the 17 shared pathways between HNASH2 and guinea pigs, supporting effects on hepatic lipid turnover as a factor in disease development. To delineate specific genes for advanced disease, leading edge genes for each dataset were reviewed (Supplementary Table S4, Additional File S1). *THRSP* was highly downregulated in the guinea pig and HNASH2 datasets compared to HNASH1, indicating a specific role for THRSP in advanced disease. Decreased THRSP serum levels is reported in patients with metabolic syndrome (increased BMI, HbA1c, triglycerides, alanine-transaminase, and lower HDL-C) compared to healthy individuals, supporting a differential regulation of THRSP when (lipid) metabolism is altered; however, hepatic histopathological status was not recorded, preventing cross-referencing to NASH [48]. Thus, increased expression in the HNASH1 dataset and decreased expression in HNASH2 and guinea pigs, and lower serum THRSP levels in patients with metabolic syndrome could indicate that THRSP varies with disease state and stimuli, and might be upregulated in mild disease, but downregulated in advanced disease. Accordingly, THRSP could be a marker of advanced NASH and would be interesting to assess as a serum marker in patients with advanced NASH. Genes involved in, e.g., steatosis-promoting pathways may be overlooked in the HNASH2 dataset, as both patient groups displayed similar degrees of hepatic steatosis. Furthermore, both patients groups were obese (BMI > 30), whereas the guinea pigs analysed in this study did not differ in body weight, also differing from the murine models, in which all Western diet-fed groups had significantly higher body weight compared to that of the controls. Regarding data analysis, only this study considered human orthologues for gene annotation of the animal model, which might also account for some of the similarity between guinea pig and human datasets.

Mitochondrial β-oxidation is central for hepatic lipid metabolism, and mitochondrial dysfunction is considered to be a symptom of advanced NASH [49,50]. Accordingly, complex 1 biogenesis and respiratory electron transport were downregulated in humans (HNASH2), guinea pigs and DIAMOND mice that also have advanced disease, indicating an overlap in late-stage pathogenesis between these models and humans [29,32]. The protein–protein association network demonstrated either the down- or no regulation of key genes involved in mitochondrial β-oxidation, including *CPT1A, PPARA,* and *ACOX1* in guinea pigs with NASH. Compared to HNASH1 patients, the advanced NASH guinea pig showed downregulation of other mitochondrial processes, i.e., peroxisome and oxidative phosphorylation. Increased mitochondrial activity was reported in patients with mild disease, similar to in the HNASH1 dataset [51]. This could be a compensatory mechanism to mitigate hepatic lipid overload by increasing fatty acid oxidation, which, over time, results in increased levels of oxidative stress and ultimately reduces mitochondrial oxidative capacity, as reported in patients with advanced disease [49]. Thus, the different stages of disease (mild vs. advanced) are likely to account for differences between HNASH1 and guinea pig expression patterns.

Within the group of inflammatory response genes HNASH2, guinea pig and murine models showed upregulated expression patterns. Closer inspection of the genes in leading edge analysis (Supplementary Table S3, Additional File S1 (highlighted in yellow)) revealed that *CCL20* and *CD44* were upregulated in HNASH2 and guinea pigs, but not in HNASH1. CCL20 is a strong chemoattractant for lymphocytes and the main ligand of the chemokine receptor CCR6, and is expressed by cholangiocytes, Kupffer cells, hepatocytes, and hepatic stellate cells [52,53]. Differential expression analysis in healthy individuals and NASH patients with lobular inflammation showed *CCL20* to be among the top 20 genes with the highest fold-change levels [54]. Furthermore, increased serum levels of CCL20 were found in NASH patients with severe fibrosis compared to those of healthy individuals [52]. In addition, the serum levels of soluble CD44 were increased in patients with NASH (*n* = 39) vs. non-NASH (*n* = 25) [55]. CD44 plays a major role in hepatic leukocyte recruitment and infiltration [56]. *CD44* null mice showed markedly decreased hepatic macrophage and neutrophil infiltration compared to wild types in response to a methionine–choline-deficient diet, and were partially protected from inflammation compared to wild types in response to a lithogenic diet [55,57]. Thus, the increased expression of *CCL20* and *CD44* appears to be linked to inflammation in NASH. As both factors can be readily measured in serum, these proteins may be interesting as biomarker candidates.

With regards to hepatic fibrosis, 4/17 of the overlapping pathways between guinea pigs and advanced NASH patients (HNASH2) are exclusively related to fibrosis. None of the pathways overlapping between DIAMOND and advanced NASH patients are involved in fibrotic processes, whereas WD1 and WD2 showed 3/7 and 12/17 of overlapping pathways, respectively. In the principal-component analysis, guinea pigs and WD1 were clearly separated by the DEGs from patients with advanced vs. mild disease (HNASH2), whereas the WD2 and DIAMOND datasets did not separate as clearly. This analysis could well be confounded by factors within the individual experiments; however, these results may collectively indicate that the fibrotic signalling network in the DIAMOND model is different from the human network, or less regulated than that in guinea pigs or WD mice. Extracellular-matrix organisation is also upregulated in DIAMOND mice, though not included in the 50, as is the case for the human and guinea pig dataset. With regards to oxidative capacity and fibrosis signalling, the guinea pig model seems to mirror the human NASH transcriptome to a higher degree than the other included preclinical models. The two human datasets include either patients with mild disease (HNASH1) or more severe NASH (HNASH2), but no healthy controls. Thus, to confirm if these findings are consistent in more progressive NASH with increased fibrosis, a comparison is warranted between the guinea pig transcriptome and an advanced NASH patient group compared to a matched healthy control. In line with the ability to display several of the human histopathological hallmarks of NASH (including fibrosis), the current findings demonstrate a clear advantage of the guinea pig model. A relatively novel preclinical model of this disease, the currently disclosed transcriptome supports a high degree of translational validity, putatively enforcing increased predictability of findings between guinea pig NASH and human patients. In this aspect, potential challenges with applying the guinea pig model (e.g., different species preferences and requirements compared to mice and rats) are outweighed. We recently reported an impact of breeder-associated variation on guinea pig NASH development [58]. Consequently, there could be differences in the NASH transcriptome between animals from different breeders, rendering the presented findings limited to guinea pigs bred at Charles River (Lyon, France).

The above findings show high similarity between guinea pig and human fibrotic gene expression, prompting further investigation of specific targets, with high clinical potential. This yielded a list of nine fibrosis-related genes, of which two secreted factors, von Willebrand factor (*VWF*) and serpin family B member 9 (*SERPINB9*), showed high correlation with the relative fibrosis area. vWF is secreted from endothelial cells, and circulating levels of vWF predicted mortality and risk of decompensation in patients with cirrhosis [59,60,61]. Furthermore, vWF increases with fibrosis stage in hepatitis C and NASH patients, supporting this as a potential marker of advancing hepatic fibrosis [42,62]. Increased *SERPINB9* expression was also reported in patients with hepatocellular carcinoma [63]. SERPINB9 could be a circulating biomarker for cytomegalovirus infection, and immunostainings confirmed the hepatocyte expression of SERPINB9 in cirrhotic hepatitis C patients [64,65]. Several of the other identified genes encode proteins that indirectly regulate the release of soluble factors to the bloodstream. This includes rhomboid 5 homolog 1 (RHBDF1), which has the highest overall correlation coefficient and regulates the activity of ADAM metallopeptidase domain 17 (ADAM17), which in turn regulates the release of tumour necrosis factor-α (TNF-α) [66]. Thus, RHBDF1 indirectly mediates the detachment of surface molecules, including TNF-α, known to contribute NASH progression, which was also among the 60 genes in common between guinea pigs and NASH patients identified from the DISEASE database [66,67,68]. Only four of the nine genes correlating with relative fibrosis area have been investigated, to the best of our knowledge, in relation to NASH. Consequently, the remaining five genes sharing a high correlation to hepatic fibrosis area and a similar expression pattern in patients with advanced NASH may serve as putative biomarkers worthy of future investigation.

## 5. Conclusions

This study showed significant overlap between the transcriptomes of the guinea pig NASH model and NASH patients with advanced fibrosis on a pathway and single-gene level. In addition to similarities in liver histopathology, this further establishes the guinea pig as a model of fibrotic NASH with high translational validity. Moreover, several genes correlating with the amount of hepatic fibrosis in guinea pigs displayed a similar expression pattern in NASH patients, supporting the clinical potential of using the guinea pig as a model in the search for biomarkers of NASH and NASH-associated fibrosis.

## Figures and Tables

**Table 1:** Overview of included preclinical models.

Preclinical Model	Sex	Species: Strain	Weeks on Diet	Histological Phenotype
Guinea pig	Female	Guinea pig: Dunkin-Hartley	25	NASH with fibrosis. Histological scoring: NASH CRN [10]
DIAMOND [32]	Male	Mouse: B6/129 (isogenic cross between C57BL/6J and 129S1/SvImJ)	52	NASH with fibrosis Histological scoring: NASH CRN [10]
WD1 [30]	Male	Mouse: C57BL/6N	12	NAFLD No histological scoring. Positive α-sma liver stain and picrosirius red indicative of activated hepatic stellate cells and fibrosis, respectively
WD2 [31]	Male	Mouse: C57BL/6J	20	NAFLD. No histological scoring

NAFLD: nonalcoholic fatty liver disease. NASH CRN: nonalcoholic steatohepatitis Clinical Research Network [10].

**Table 2:** Histological scoring of control and NASH guinea pigs.

Group	Control (*n* = 6)	NASH (*n* = 6)
Steatosis ^1^	0	3 **
Ballooning ^1^	0	2 (1–2) *
Inflammation ^1^	0 (0–1)	3 **
Fibrosis ^1^	0	3 **
Relative fibrosis area ^2^	1.39 ± 0.24	7.48 ± 1.81 ***

Steatosis, Ballooning, inflammation, and fibrosis: medians with range (if applicable). Relative fibrosis area: means with standard deviations. Histopathological scoring data (steatosis, ballooning, inflammation, and fibrosis) is previously published [6]. ^1^ Analysed using Mann–Whitney U test. ^2^ Analysed by unpaired t-test with Welch’s correction. * *p* < 0.05, ** *p* < 0.01 and *** *p* < 0.001.

**Table 3:** Top 20 differentially expressed genes in guinea pigs.

Gene Name (Full Name)	Log2 Fold Change	Adjusted *p*-Value	Function
*ADAMDEC1* (ADAM like decysin 1)	8.01	4.94 ×10^−60^	Secreted protein invovled in dendritic cell maturation.
*ADGRG3* (adhesion G protein-coupled receptor 3)	7.66	3.79 × 10^−40^	GPCR possibly invovled tumor angiogenesis.
*KRT23* (keratin 23)	10.97	1.53 × 10^−35^	Member of keratin family of intermediate filaments responsible for structural integrity of epithelial cells.
*ATP6V0A4* (ATPase H + transporting V0 subunit a4)	9.02	3.01 × 10^−32^	Vacuolar ATPase mediating acidification of intracellular compartments necessary for protein sorting, zymogen activation, receptor-mediated endocytosis and synaptic vesicle protein gradient generation.
*TMEM213* (transmembrane protein 213)	10.12	1.28 × 10^−29^	No listed function.
*CIB4* (calcium and integrin binding family member 4)	−9.70	1.82 × 10^−26^	No listed function.
*PAK6* (p21 (RAC1) activated kinase 6)	9.38	5.69 × 10^−26^	p21 stimulated serine/threonine kinase involved in cytoskeleton rearrangement, apoptosis and MAP kinase signalling pathway.
*TMC1* (transmembrane channel like 1)	9.27	1.82 × 10^−25^	No listed function.
*CCL7* (C–C motif chemokine ligand 7)	9.09	2.30 × 10^−25^	Encodes MCP3-a secreted chemokine recruiting macrophages during inflammation, and also a substrate of MMP2.
*PTPRN* (protein tyrosine phosphatase receptor type N)	9.23	2.58 × 10^−24^	Signalling molecule regulating processes such as cell growth, differentiation, mitotic cycle, and oncogenic transformation.
*VSIG1* (V-set and immunoglobulin domain containing 1)	8.26	1.06 × 10^−22^	Encodes a member of the junctional adhesion molecule (JAM) family.
*SLC34A2* (solute carrier family 34 member 2)	8.49	1.13 × 10^−20^	pH-sensitive sodium-dependent phosphate transporter
*DSG4* (desmoglein 4)	8.15	2.78 × 10^−19^	Desmosomal cadherin possibly playing a role in cell–cell adhesion in epithelial cells.
*TNFSF18* (TNF super family member 18)	7.74	1.92 × 10^−17^	Cytokine belonging to the TNF ligand family that plays a role in T-lymphocyte survival and the interaction between endothelial cells and T lymphocytes.
*MTHFD2* (methylenetetrahydrofolate dehydrogenase (NADP + dependent) 2, methenyltetrahydrofolate cyclohydrolase)	7.63	6.53 × 10^−17^	Nuclear encoded mitochondrial bifunctional enzyme with methylenetetrahydrofolate dehydrogenase and methenyltetrahydrofolate cyclohydrolase activities.
*SPOCK1* (SPARC (osteonectin), cwcv and kazal-like domains proteoglycan 1)	7.72	8.38 × 10^−17^	Seminal plasma proteoglycan containing chondroitin and heperan sulfate chains.
*ECT2* (epithelial cell transforming 2)	8.43	5.94 × 10^−16^	Guanine nucleotide exchange factor, expressed at high levels in mitotic cells in the regenerating liver.
*SPTA1* (spectrin alpha, erythrocytic 1)	7.99	7.35 × 10^−15^	Molecular scaffold protein that links the plasma membrane to the actin cytoskeleton and determines the cell shape.
*STAR* (steroidogenic acute regulatory protein)	8.36	2.48 × 10^−14^	Involved in the acute regulation of steroid hormone synthesis by enhancing the conversion of cholesterol into pregnolone.
*KEL* (Kell metalloendopeptidase (Kell blood group))	7.66	1.34 × 10^−12^	Encodes a type II transmembrane glycoprotein of the Kell blood group antigen.

List of top 20 differentially expressed genes in the guinea pig dataset based on log2 fold change and Benjamini–Hochberg adjusted *p*-value. GPCR: G protein-coupled receptor, MAP: mitogen-activated protein, MCP: monocyte chemotactic protein, MMP: matrix metalloproteinase, TNF: tumour necrosis factor.

**Table 4:** Genes related to fibrosis quantification.

Gene	Pearson’s ρ	*p* Value	GPLog2FC	HLog2FC	Function ^1^	Secreted	Role in NASH	Cell-Specific Expression ^2^
*ACKR3*	All: 0.91 NASH: 0.87	All: 4.54 × 10^−5^ NASH: 0.025	1.36	HNASH1: ND HNASH2: 0.69	GPCR, orphan receptor	NO	?	Endothelial cells
*BIRC3*	All: 0.88 NASH: 0.82	All: 1.77 × 10^−4^ NASH: 0.045	1.1	HNASH1: 1.9 HNASH2: 0.7	Inhibits apoptosis	NO	YES (hypoxia induced) [39]	Immune cells, cholangiocytes, endothelial cells, and hepatocytes
*CHST11*	All: 0.95 NASH: 0.87	All: 1.42 × 10^−6^ NASH: 0.023	1.16	HNASH1: −0.35 HNASH2: 0.19	Promotes synthesis of chondroitin (ECM)	NO	?	Immune cells
*EMP3*	All: 0.93 NASH: 0.81	All: 9.41 × 10^−6^ NASH: 0.049	1.5	HNASH1: 0.98 HNASH2: 0.19	Membrane protein, cell proliferation	NO	?	Immune cells
*FZD7*	All: 0.87 NASH: 0.83	All: 2.22 × 10^−4^ NASH: 0.041	1.2	HNASH1: ND HNASH2: 0.6	Wnt signalling	NO	YES in HCC [40]	Cholangiocytes, HSC
*RGS14*	All: −0.83 NASH: −0.80	All: 8.26 × 10^−4^ NASH: 0.053	−1.58	HNASH1: −0.3 HNASH2: −0.2	Regulates GPCR (increases microtubule assembly)	NO	?	Immune cells
*RHBDF1*	All: 0.96 NASH: 0.92	All: 4.11 × 10^−7^ NASH: 0.010	1.17	HNASH1: 0.6 HNASH2: 0.03	Regulates ADAM17 and release of TNF-α	NO	?	Cholangiocytes
*SERPINB9*	All: 0.9 NASH: 0.84	All: 6.19 × 10^−5^ NASH: 0.037	1.4	HNASH1: −0.7 HNASH2: 0.5	Inhibits activity of granzyme B	YES	YES [41]	Immune cells, endothelial cells, stellate cells, and myofibroblasts, macrovascular endothelial cells
*VWF*	All: 0.97 NASH: 0.86	All: 7.52 × 10^−8^ NASH: 0.027	1.46	HNASH1: −0.03 HNASH2: 0.5	Platelet aggregation	YES	YES [42,43]	Macrovascular endothelial cells

Genes listed in alphabetical order. In Pearson’s ρ column: All, correlation calculated using both control and NASH animals; NASH, correlation calculated using only NASH animals. ^1^ Description of function based on [44]. ^2^ Based on liver cell atlas [45,46]. ECM: extracellular matrix, GPCR: G-protein coupled receptor, GPLog2FC: guinea pig log2 fold change, HCC: hepatocellular carcinoma, HLog2FC: human log2 fold change, HSC: hepatic stellate cell, HNASH1: NASH patients with mild fibrosis vs. healthy controls. HNASH2: NASH patients with advanced fibrosis vs. NASH patients with no or mild fibrosis.

## Data Availability

The generated and analysed dataset from the current study is available at www.ncbi.nlm.nih.gov/geo, geo accession number GSE158168. The analysed datasets are available at www.ncbi.nlm.nih.gov/geo, accession numbers GSE52748, GSE38141, GSE67680, GSE126848, and GSE49541.

## Supplementary Materials

The following are available online at https://www.mdpi.com/article/10.3390/biomedicines9091198/s1. Additional File S1: Word document docx. Supplementary Table S1. Table of relative fibrosis area based on Picro Sirius red staining for each animal. Supplementary Table S2. List of top 50 overlapping pathways between the preclinical murine models and HNASH2 dataset. Supplementary Table S3. List of all Hallmark gene sets for the guinea pig dataset, including leading edge genes. Supplementary Table S4. List of Reactome pathway gene set: fatty acid metabolism for HNASH1, HNASH2, and guinea pig. Additional File S2: PDF of linear regression on the normalised (normalised by DeSeq2 size factor) and rlog-transformed values, and the fibrosis fraction for each animal, for each of the nine genes identified in correlation analysis. R^2^ and the linear equation are reported for each gene.
