# Supplementary material for: Modelling Nonalcoholic Steatohepatitis In Vivo—A Close Transcriptomic Similarity Supports the Guinea Pig Disease Model
Source: Biomedicines. 2021 Sep 10;9(9):1198. doi: 10.3390/biomedicines9091198 (PMC8471870; doi:10.3390/biomedicines9091198)
Supplement: Supplementary file 1 [file biomedicines-09-01198-s001.zip › biomedicines-1360335-supplementary.pdf]

Supplementary Materials

Supplementary Table S1. Fibrosis fraction for each animal in %.

ID	Group	Fibrosis fraction [%]
5	Control	1.718
10	Control	1.203
31	Control	1.358
32	Control	1.097
45	Control	1.322
56	Control	1.644
2	NASH	10.413
9	NASH	7.925
11	NASH	6.219
20	NASH	5.700
41	NASH	6.162
65	NASH	8.480

Table S2. Normalized enrichment scores for the Reactome pathways in common between preclinical models and HNASH2 dataset. See fig. 4F for a graphical overview of guinea pig and HNASH2 pathways. NES: normalized enrichment score. HNASH2: GSE49541, DIAMOND: GSE67680, WD1: GSE52748, WD2: GSE38141.

Pathway	HNASH2 NES	DIAMOND NES	WD1 NES	WD2 NES	Guinea pig
COMPLEX 1 BIOGENESIS	-1.9	-1.9			-2.3
RESPIRATORY ELECTRON TRANSPORT	-1.9	-1.9			-2.6
RESPIRATORY ELECTRON TRANSPORT ATP SYNTHESIS BY CHEMIOSMOTIC COUPLING AND HEAT PRODUCTION BY UNCOULING PROTEINS	-1.9	-1.8			-2.5
THE CITRIC ACID TCA CYCLE AND RESPIRATORY ELECTRON TRANSPORT	-1.9	-1.7			-2.4
CHEMOKINE RECEPTORS BIND CHEMOKINES	1.8	1.9			
GLYOXYLATE METABOLISM AND GLYCINE DEGRADATION	-1.9	-1.9			-2.1
MITOCHONDRIAL TRANSLATION	-2.0	-1.6			-2.4
METABOLISM OF AMINO ACIDS AND DERIVATIVES	-2.1	-1.8	-2.2		
BIOLOGICAL OXIDATIONS	-1.9		-1.7		
MITOCHONDRIAL FATTY ACID BETA OXIDATION	-1.8		1.9		
FATTY ACID METABOLISM					-2.0
CHOLESTEROL BIOSYNTHESIS	-1.8	-2.3	-2.5	-2.6	-2.6
PEROXISOMAL LIPID METABOLISM					-2.5
PEROXISOMAL PROTEIN IMPORT					-2.6
ECM PROTEOGLYCANS	2.1		1.8	2.2	1.9
ASSEMBLY OF COLLAGEN FIBRILS AND OTHER MULTIMERIC STRUCTURES	1.9		1.7	2.0	
COLLAGEN FORMATION	1.8		1.7	2.0	
CLASS A 1 RHODOPSIN LIKE RECEPTORS	1.7			1.5	
COLLAGEN CHAIN TRIMERIZATION	1.9			1.9	
COLLAGEN DEGRADATION	1.9			1.9	
COLLAGEN BIOSYNTHESIS AND MODIFYING ENZYMES	1.8			2.0	
ELASTIC FIBRE FORMATION	1.8			1.9	1.9
INTEGRIN CELL SURFACE INTERACTIONS	2.9			2.1	
SIGNALING BY PDGF	1.8			1.9	
DEGRADATION OF THE EXTRACELLULAR MATRIX	2.0			1.9	
EXTRACELLULAR MATRIX ORGANIZATION	2.0			2.1	1.9
NON-INTEGRIN MEMBRANE ECM INTERACTIONS	1.9			2.0	
MOLECULES ASSOCIATED WITH ELASTIC FIBRES	1.7			1.9	1.9
O GLYCOSYLATION OF TSR CONTAINING MOLECULES	1.8			1.8	
MET ACTIVATES PTK2 SIGNALING	1.8			1.9	
NCAM SIGNALING FOR NEURITE OUT GROWTH					1.7
PHASE I FUNCTIONALIZATION OF COMPOUNDS					-2.1
PROTEIN LOCALIZATION					-2.5

Supplementary Table S3. Hallmark gene set in guinea pig, including leading edge genes. Genes included in the manuscript are highlighted (in yellow). Pval: p-value, Padj: p-adjusted, ES: enrichment score, NES: normalized enrichment score.

Pathway	Pval	Padj	ES	NES	Leading Edge
**TNFA SIGNALING VIA NFKB**	0.000103	0.000862	0.663162	1.923282	CCL2 , SERPINE1 , CCL20 , ABCA1 , CCN1 , EGR2 , EGR3 , TNFAIP6 , PFKFB3 , CCL4 , TNFAIP2 , NR4A3 , MYC , CD83 , SLC2A6 , GPR183 , DUSP4 , GFPT2 , CLCF1 , RELB , IL18 , GADD45A , KLF6 , JAG1 , SLC16A6 , NR4A1 , F2RL1 , IL7R , EGR1 , PHLDA2 , TLR2 , NFKBIE , SPSB1 , CDKN1A , LIF , PLAU , TNF , FOSL1 , CD44 , IFNGR2 , NFKB2 , DRAM1 , OLR1 , ATF3 , ACKR3 , SQSTM1 , PHLDA1 , TRAF1 , RCAN1 , DUSP2 , F3 , FOSL2 , PLEK , TNFAIP3 , SOD2 , BIRC3 , CCND1 , RNF19B , EDN1 , TRIP10 , BCL3 , ZC3H12A , PLAUR , GEM , TIPARP , SMAD3 , BTG3 , PTGER4 , ETS2 , BTG1
**HYPOXIA**	0.014579	0.028037	0.475028	1.384494	SERPINE1 , CCN5 , CCN1 , PFKFB3 , DTNA , TGFB3 , S100A4 , PPFIA4 , COL5A1 , SLC6A6 , PDGFB , CSRP2 , STC2 , TMEM45A , AMPD3 , STC1 , KLF6 , PLIN2 , CXCR4 , HK2 , CHST3 , PGM2 , CDKN1A , TPBG , SDC3 , TES , ATF3 , ACKR3 , NAGK , SRPX , PFKP , GPC1 , F3 , PGF , FOSL2 , CAVIN3 , PIM1 , TNFAIP3 , CAVIN1 , PDK3 , PLAUR , ALDOA , MYH9 , TIPARP , KIF5A , HS3ST1 , CASP6 , STBD1 , BTG1 , WSB1 , HMOX1 , NDRG1 , CCN2 , MAP3K1 , LXN , ENO3
**CHOLESTEROL HOMEOSTASIS**	0.559884	0.651027	0.352277	0.957999	FABP5 , LGALS3 , LPL , TNFRSF12A , FADS2 , ANXA5 , CD9 , FASN , S100A11 , JAG1 , CHKA , ATF3 , GPX8 , CLU , PDK3 , PLAUR
**MITOTIC SPINDLE**	0.000102	0.000862	0.634516	1.852352	ECT2 , KIF2C , DLGAP5 , CCNB2 , ANLN , BUB1 , KIF15 , KIF4A , NDC80 , TOP2A , CENPE , FBXO5 , RACGAP1 , PRC1 , KIF11 , NEK2 , TPX2 , KIF22 , SASS6 , CD2AP , SYNPO , CENPF , RHOF , ESPL1 , LLGL1 , CDC42EP1 , GSN , PIF1 , KIF20B , BIRC5 , DOCK2 , SPTAN1 , ABR , EZR , BCR , ARHGEF2 , NEDD9 , INCENP , ARHGAP27 , FSCN1 , EPB41L2 , FLNA , MYO9B , PREX1 , NF1 , PLEKHG2 , MYH9 , NIN , MID1IP1 , LMNB1 , KIF3C , ARHGEF3 , PCNT , VCL , RICTOR , MYO1E , DYNC1H1 , PKD2 , ALMS1 , EPB41 , CDC42BPA , BCAR1 , HOOK3 , ARL8A , KNTC1 , ARF6 , SORBS2 , OPHN1 , ARHGAP10 , ARHGDIA , KIF5B
**WNT BETA CATENIN SIGNALING**	0.486469	0.599236	0.412288	0.998731	MYC , JAG1 , CSNK1E , WNT5B , PPARD , TP53 , HEY1 , ADAM17 , NKD1 , RBPJ
**TGF BETA SIGNALING**	0.15787	0.246125	0.48534	1.256362	LTBP2 , THBS1 , SERPINE1 , SKIL , IFNGR2 , TGFB1 , WWTR1 , HDAC1 , SMAD3 , PMEPA1 , XIAP , HIPK2 , SLC20A1 , CDH1
**IL6 JAK STAT3 SIGNALING**	0.003368	0.010524	0.591213	1.603562	CCL7 , TNFRSF12A , ACVR1B , ITGA4 , TNFRSF21 , CD9 , TLR2 , TNF , CD44 , CCR1 , ITGB3 , IFNGR2 , TNFRSF1B , CSF3R , TGFB1 , PIM1 , BAK1 , CNTFR , IL12RB1 , ACVRL1 , OSMR , HMOX1 , MAP3K8 , IL10RB , IL17RA , CD36 , TNFRSF1A , STAT1 , PTPN1 , IFNGR1 , PTPN11 , IL13RA1 , IL2RG , IL4R , FAS , IL1B , IL6ST
**DNA REPAIR**	0.977244	0.977244	0.222308	0.640852	RAD51 , TYMS , CDA , TAF1C , HCLS1 , BCAM , ZWINT , POLD1 , REV3L , POLA2 , ADA , RFC4 , VPS37B , RALA , NPR2 , AGO4 , CMPK2 , VPS37D , SMAD5 , TP53 , POLA1 , RFC5
**G2M CHECKPOINT**	0.000103	0.000862	0.723423	2.111434	KIF2C , CCNB2 , CDC45 , CDC20 , BUB1 , KIF15 , KIF4A , NDC80 , EXO1 , TOP2A , CDC6 , TROAP , CCNA2 , AURKB , POLE , CENPE , CENPA , FBXO5 , UCK2 , RACGAP1 , RAD54L , PRC1 , KNL1 , KIF11 , PTTG1 , SLC38A1 , HMMR , PBK , NEK2 , HMGB3 , MCM2 , MYC , TACC3 , TPX2 , MYBL2 , KIF22 , MCM3 , TRAIP , BARD1 , STMN1 , CKS2 , CHAF1A , CENPF , ESPL1 , SLC7A5 , E2F2 , CDKN3 , CDKN2C , KIF20B , BIRC5 , SLC7A1 , SMC2 , TGFB1 , MCM6 , NSD2 , INCENP , CCND1 , CDC25A , CDC7 , CHEK1 , PRIM2 , BCL3 , ODC1
**APOPTOSIS**	0.010007	0.020849	0.496262	1.428288	LGALS3 , TOP2A , TNFRSF12A , EMP1 , ANXA1 , EGR3 , IGFBP6 , TIMP1 , MMP2 , IL18 , GADD45A , PAK1 , PDGFRB , CDKN1A , PLCB2 , GSN , TNF , CD44 , TIMP2 , HMGB2 , ATF3 , SQSTM1 , SPTAN1 , CASP4 , GPX3 , KRT18 , PMAIP1 , LUM , NEDD9 , CLU , SOD2 , BIRC3 , CCND1 , ERBB2 , BCL2L1 , TGFB2 , IGF2R , BTG3 , CASP6 , WEE1 , HMOX1 , ANKH , CASP2 , CDC25B , CASP8 , CFLAR
**NOTCH SIGNALING**	0.257795	0.368278	0.492352	1.184663	JAG1 , DTX4 , MAML2 , ARRB1 , FZD7 , HEYL , NOTCH3 , CCND1 , PRKCA , PPARD , FBXW11 , NOTCH2 , WNT5A , KAT2A
**ADIPOGENESIS**	0.004348	0.01087	-0.32836	-1.41819	CIDEA , DHCR7 , GPAM , PHYH , MARC2 , ITGA7 , FAH , ARL4A , ACOX1 , PIM3 , TOB1 , ATL2 , SOD1 , CAT , TST , DDT , QDPR , FZD4 , ECHS1 , DECR1 , CMBL , DHRS7 , ALDH2 , REEP6 , HADH , CYP4B1 , LIPE , ETFB , ACADS , MGLL , SLC25A1 , ACADM , SORBS1 , PRDX3 , PPP1R15B , RIOK3 , DBT , POR , NDUFA5 , SDHC , CAVIN2 , DNAJB9 , UQCR11 , IDH1 , CRAT , ACAA2 , CHCHD10 , AIFM1 , COX6A1 , ANGPTL4 , GRPEL1 , ATP5PO , PGM1 , NDUFB7 , NKIRAS1 , MGST3 , NDUFS3 , COX7B , HIBCH , DHRS7B , GPX4 , GPAT4 , AK2 , UQCR10 , DRAM2 , SUCLG1 , COQ5 , MCCC1 , STOM , NDUFAB1 , COX8A , PFKL , GBE1 , MDH2 , ADIPOR2 , SLC19A1 , UQCRC1 , ESRRA , COQ3 , UBQLN1
**ESTROGEN RESPONSE EARLY**	0.066028	0.110047	0.437948	1.274314	CLDN7 , SLC1A4 , CCN5 , MREG , THSD4 , MYOF , GAB2 , EGR3 , INHBB , OLFML3 , HR , GJA1 , MYC , IGF1R , ANXA9 , STC2 , NPY1R , ZNF185 , FASN , SFN , SLC7A5 , FHL2 , TPBG , SLC26A2 , KRT19 , CD44 , ALDH3B1 , NAV2 , LAD1 , SYT12 , KRT8 , KRT18 , CISH , P2RY2 , PMAIP1 , ELF3 , TGIF2 , CCND1 , ABLIM1 , FCMR , WWC1 , MLPH , TIPARP , CBFA2T3
**ESTROGEN RESPONSE LATE**	0.047143	0.086003	0.448442	1.30248	KIF20A , CDC20 , FABP5 , TOP2A , CDC6 , SLC1A4 , CCN5 , MYOF , EGR3 , ST14 , LAMC2 , CPE , MDK , CA2 , HR , AGR2 , SLC2A8 , MAPK13 , ANXA9 , CD9 , TFPI2 , NPY1R , ATP2B4 , S100A9 , SFN , SLC7A5 , TPBG , SLC26A2 , KRT19 , CD44 , ALDH3B1 , DLG5 , NAB2 , CISH , GJB3 , DUSP2 , RNASEH2A , LTF , CCND1 , CKB
**ANDROGEN RESPONSE**	0.7539	0.802021	0.304097	0.844261	ABCC4 , PTK2B , FADS1 , MAK , CENPN , SLC26A2 , KRT19 , KRT8 , CDK6 , LIFR , CCND3 , CCND1 , ITGAV , AKT1 , SRF , NDRG1 , ANKH , ACTN1 , PMEPA1 , STK39 , MYL12A , RRP12 , MERTK , PGM3 , SEC24D
**MYOGENESIS**	0.002085	0.007446	0.528806	1.521953	ACHE , SLC6A8 , COL1A1 , COL6A3 , DTNA , BDKRB2 , PPFIA4 , COL4A2 , COL6A2 , APOD , STC2 , AEBP1 , ITGB4 , COL3A1 , KLF5 , MAPK12 , CDKN1A , PTGIS , GSN , TNNI2 , ATP2A1 , SPARC , LAMA2 , NAV2 , SPTAN1 , GPX3 , TGFB1 , PFKM , FABP3 , SOD3 , CLU , SORBS3 , WWTR1 , IGFBP7 , CFD , ABLIM1 , AGRN , CKB , CACNA1H , SSPN , EFS , MYH9 , FST , RYR1 , ITGB5 , EPHB3 , TNNT1
**PROTEIN SECRETION**	0.873435	0.899727	0.268708	0.75276	ABCA1 , SCRN1 , TSPAN8 , CD63 , KRT18 , ICA1 , SSPN , IGF2R , ADAM10 , CLCN3 , VAMP3 , GLA , ARFGEF2 , SEC24D , RAB22A , YKT6 , RPS6KA3 , KIF1B , ARFGEF1 , STX7 , DOP1A , GALC , SNAP23 , STAM , COG2
**INTERFERON ALPHA RESPONSE**	0.58259	0.662034	0.343797	0.945845	LY6E , MX1 , SAMD9 , IFI30 , RSAD2 , ISG15 , SAMD9L , IRF7 , DDX60 , LGALS3BP , SELL , CASP8 , PARP14 , OASL , CD74 , ISG20 , CMPK2 , ELF1 , UBE2L6 , IL4R , CD47 , PARP12 , CASP1 , DHX58 , BATF2
**INTERFERON GAMMA RESPONSE**	0.008578	0.018648	0.492534	1.428432	CCL7 , MTHFD2 , CCL2 , TNFAIP6 , TNFAIP2 , UPP1 , OAS2 , CDKN1A , LY6E , CD86 , MX1 , IFI30 , SAMHD1 , CD274 , CMKLR1 , FGL2 , CASP4 , SLAMF7 , BPGM , STAT4 , PFKP , LYSMD2 , RSAD2 , ST8SIA4 , PIM1 , TNFAIP3 , SOD2 , IL10RA , ISG15 , SAMD9L , SELP , CIITA , SSPN , IDO1 , IRF7 , DDX60 , LGALS3BP , VAMP8 , BTG1 , OAS3 , LCP2 , CASP8 , ITGB7 , PARP14 , OASL , IRF4 , CD74 ,

					ISG20 , CMPK2 , STAT1 , PML , NOD1 , PTPN1 , ZNFX1 , ST3GAL5 , RIPK1 , NUP93 , UBE2L6 , PSMB10 , IL4R , FAS , IL18BP , NFKB1 , PTPN6 , VAMP5 , CSF2RB , HIF1A , PARP12 , CD40 , CASP1 , RAPGEF6 , JAK2 , DHX58 , ZBP1 , BATF2 , PLA2G4A , NFKBIA , ARID5B
**APICAL JUNCTION**	0.001245	0.005186	0.534529	1.544501	CLDN7 , COL16A1 , CLDN4 , LAMC2 , MDK , THBS3 , MAPK13 , CD276 , ITGA3 , ZYX , MMP2 , ITGB4 , FLNC , RHOF , SDC3 , SYK , ADRA1B , CNN2 , NRTN , CD86 , CX3CL1 , VWF , NECTIN2 , SRC , YWHAH , CD274 , CD34 , VASP , TRAF1 , RRAS , TMEM8B , SORBS3 , FSCN1 , EPB41L2 , LAYN , NF1 , ACTB , MYH9 , EVL , BAIAP2 , ADAM9 , ARHGEF6 , PTPRC , FYB1 , CTNNA1 , VCL , MAPK11 , GNAI2 , MSN , ACTN1 , SLIT2 , NECTIN1
**APICAL SURFACE**	0.484276	0.599236	0.407368	1.001239	ATP6V0A4 , PKHD1 , SULF2 , SLC2A4 , CX3CL1 , AFAP1L2 , SRPX , DCBLD2 , TMEM8B , PLAUR
**HEDGEHOG SIGNALING**	0.048162	0.086003	0.622638	1.457868	ACHE , NRP2 , CDK6 , ADGRG1 , CNTFR , NF1 , MYH9 , DPYSL2 , ETS2 , OPHN1 , PML , HEY1
**COMPLEMENT**	0.00361	0.010618	0.504725	1.466506	MMP12 , LGALS3 , SERPINE1 , CPM , PRSS36 , CA2 , COL4A2 , PDGFB , ME1 , FCN1 , ANXA5 , TIMP1 , TFPI2 , DOCK9 , S100A9 , MT3 , ITGAM , CDA , CTSH , TIMP2 , SRC , PIK3CG , LIPA , CTSD , PRKCD , OLR1 , CASP4 , CASP5 , ZFPM2 , PLA2G7 , F3 , CTSB , CLU , PLEK , PIM1 , LTF , TNFAIP3 , FCER1G , CTSS , GPD2 , CTSV , WAS , PLAUR , MMP15 , CSRP1 , IRF7 , DOCK10 , ADAM9 , GCA , GNB4 , KIF2A , CBLB , PRCP , GNAI2 , LCP2
**UNFOLDED PROTEIN RESPONSE**	0.052108	0.089841	0.474062	1.336823	MTHFD2 , DNAJA4 , CHAC1 , CCL2 , SLC1A4 , STC2 , SLC7A5
**PI3K AKT MTOR SIGNALING**	0.24373	0.358426	0.414875	1.151043	RPS6KA1 , CXCR4 , SFN , MAP2K6 , CDKN1A , VAV3 , PRKCB , SLA , SQSTM1 , AKT1 , THEM4 , ACTR3 , RALB , TNFRSF1A , NOD1 , ARHGDIA , PTPN11 , RIPK1 , SMAD2 , IL2RG , DAPP1 , RPS6KA3 , NCK1
**MTORC1 SIGNALING**	0.491373	0.599236	0.343093	1.004857	MTHFD2 , RRM2 , BUB1 , FADS2 , SLC1A4 , MCM2 , SLC6A6 , ME1 , CD9 , NUPR1 , FADS1 , SLC1A5 , STC1 , CXCR4 , G6PD , HK2 , SLC7A5 , CDKN1A , EGLN3 , TES , SLA , IFI30 , SERPINH1 , FGL2 , SQSTM1
**E2F TARGETS**	0.000102	0.000862	0.716191	2.093167	MTHFD2 , KIF2C , DLGAP5 , CDCA3 , CCNB2 , UBE2T , RRM2 , CDC20 , TRIP13 , KIF4A , DEPDC1 , CDCA8 , TOP2A , AURKB , POLE , CENPE , MELK , RACGAP1 , TK1 , PTTG1 , DCK , GINS4 , CCNE1 , HMMR , HMGB3 , MCM2 , MYC , WDR90 , TACC3 , MYBL2 , KIF22 , SPC25 , CTPS1 , SPAG5 , MCM3 , GINS3 , BARD1 , STMN1 , CKS2 , MXD3 , ESPL1 , CDKN3 , TCF19 , CDKN1A , E2F8 , CDKN2C , TUBB , RAD51AP1 , HMGB2 , BIRC5
**MYC TARGETS V1**	0.531452	0.632681	0.338762	0.988217	CDC45 , CDC20 , CCNA2 , MCM2 , MYC , TYMS , CTPS1
**MYC TARGETS V2**	0.881733	0.899727	0.269995	0.711725	MYC , HK2 , DUSP2 , RRP9 , MCM5 , PPRC1 , MCM4 , MYBBP1A , TCOF1 , RRP12 , PLK1 , LAS1L , CBX3 , UTP20 , NOP56 , BYSL , PRMT3 , WDR43 , SRM , DDX18 , GNL3 , CDK4 , MRTO4 , TBRG4
**EPITHELIAL MESENCHYMAL TRANSITION**	0.000103	0.000862	0.67044	1.943832	SPOCK1 , SLC6A8 , COL16A1 , RGS4 , COL1A1 , THBS1 , TNFRSF12A , SERPINE1 , PMP22 , FMOD , CCN1 , LGALS1 , COL6A3 , LAMC2 , MGP , CXCL8 , LOXL1 , THBS2 , GJA1 , DPYSL3 , COL1A2 , COL8A2 , CAPG , COL4A1 , COL5A1 , COL4A2 , COL6A2 , SLIT3 , FSTL3 , TIMP1 , QSOX1 , TFPI2 , COL12A1 , MMP2 , COL3A1 , GADD45A , SPP1 , NID2 , FBLN2 , PDLIM4 , TPM1 , FBLN5 , PDGFRB , GLIPR1 , COL5A2 , CD44 , EMP3 , SPARC , LAMA2 , ITGB3 , ABI3BP , VIM , SERPINH1 , LOXL2 , EFEMP2 , TGFB1 , GPC1 , PLOD2 , LUM , LAMC1 , ACTA2 , TNFAIP3 , APLP1 , ITGAV , FLNA , PLAUR , SGCB , GEM , SERPINE2 , ITGB5 , COMP
**INFLAMMATORY RESPONSE**	0.000103	0.000862	0.609269	1.765453	CCL7 , CCL2 , HAS2 , ITGB8 , RGS1 , SERPINE1 , CCL20 , ABCA1 , TNFAIP6 , CCL22 , RGS16 , CXCL8 , ACVR1B , MYC , KCNA3 , CLEC5A , TIMP1 , GPR183 , SLC28A2 , C5AR1 , IL18 , KLF6 , IL7R , TLR2 , CDKN1A , TPBG , LY6E , LIF , CYBB , CX3CL1 , SGMS2 , EMP3 , FFAR2 , ITGB3 , IFNGR2 , CMKLR1 , OLR1 , SLC7A1 , NLRP3 , LPAR1 , TNFRSF1B , P2RY2 , CSF3R , P2RX7 , DCBLD2 , F3 , MSR1 , NOD2 , IL10RA , EDN1
**XENOBIOTIC METABOLISM**	0.004202	0.01087	-0.53052	-2.287	CYP1A2 , PTGDS , SERPINA6 , CYP26A1 , G6PC , CYP2E1 , BCAT1 , CRP , ADH1C , TTPA , RBP4 , GCH1 , FMO3 , FAH , CROT , KYNU , DCXR , ITIH1 , ACOX1 , CYP1A1 , FETUB , CAT , ARG1 , TDO2 , TAT , FMO1 , ATOH8 , DDT , IGF1 , ITIH4 , MAOA , DDC , MBL2 , GNMT , ALAS1 , MPP2 , PGRMC1 , DHRS7 , ALDH2 , IGFBP4 , ALDH9A1 , AOX1 , UPB1 , CYB5A , CSAD , ENTPD5 , TPST1 , ENPEP , HRG , LPIN2 , LCAT , BPHL , HACL1 , HSD11B1 , CDO1 , ACOX2 , F10 , IL1R1 , AHCY , IGFBP1 , F11 , POR , NFS1 , ASL , GSTO1 , TMBIM6 , NQO1 , BLVRB , VTN , IDH1 , AKR1C3 , ACP2 , RAP1GAP , SHMT2 , LEAP2 , SAR1B , PLG , GSTM4 , MTHFD1 , HGFAC , ABHD6
**FATTY ACID METABOLISM**	0.002695	0.008985	-0.51307	-2.1286	DHCR24 , CIDEA , HSD17B7 , CPOX , NSDHL , ADH1C , ALDH1A1 , GPD1 , IDI1 , HSD17B4 , ACOX1 , CYP1A1 , CRYZ , ECI2 , TDO2 , HMGCS1 , FMO1 , MAOA , GSTZ1 , ECHS1 , CPT1A , HSD17B11 , DECR1 , REEP6 , ALDH9A1 , ACSL1 , ECI1 , HMGCS2 , HADH , ACOT2 , ACAA1 , ALDH3A2 , ACADS , MGLL , BCKDHB , HPGD , ACADM , BPHL , CYP4A22 , CYP4A11 , AOC3 , SUCLG2 , HMGCL , BLVRA , NTHL1 , AUH , AADAT , ACAT2 , GRHPR , PCBD1 , SDHC , FH , SDHD , MCEE , IDH1 , GCDH , METAP1 , CRAT , ACAA2 , RDH11 , ALAD , OSTC , HIBCH , MLYCD , ETFDH , PDHB , SUCLA2 , PSME1 , SUCLG1 , D2HGDH , SLC22A5 , MDH1
**OXIDATIVE PHOSPHORYLATION**	0.005102	0.012148	-0.59473	-2.56985	PHYH , TIMM10 , ATP5MC2 , ECHS1 , SLC25A11 , CPT1A , ALAS1 , DECR1 , ALDH6A1 , ECI1 , CYB5A , ACAA1 , IDH2 , ETFB , ACADM , OAT , PRDX3 , FDX1 , GLUD1 , ETFA , SLC25A4 , NDUFC2 , MAOB , POR , NDUFV1 , NDUFA5 , SDHC , ACAT1 , SLC25A20 , FH , SDHD , UQCR11 , IDH1 , COX4I1 , NDUFA2 , NDUFA3 , ISCU , ATP5MG , COX5A , ATP1B1 , ATP5PD , ACAA2 , NDUFS2 , UQCRB , NDUFB5 , AIFM1 , COX11 , PDK4 , COX6A1 , COX6B1 , TIMM13 , GRPEL1 , COX5B , ATP5PO , MPC1 , COX7A2 , NDUFB7 , MGST3 , NDUFB6 , ATP5ME , NDUFA4 , ATP5MC1 , NDUFS3 , COX7B , NDUFB4 , NDUFS8 , GPX4 , COX7C , UQCR10 , NDUFS6 , NDUFA9 , COX6C , RHOT1 , ETFDH , ATP5PF , PDHB , SUCLA2 , NDUFS4 , SUCLG1 , MTRF1 , UQCRH , MDH1 , NDUFS7 , NDUFA7 , NDUFAB1 , HADHB , BDH2 , COX8A , MRPS15 , MDH2 , UQCRC2 , IDH3B , UQCRC1 , CYB5R3 , NDUFB8 , POLR2F , PDHA1 , ATP5F1E , MRPS12 , SDHA , ATP5PB , NDUFB3 , NDUFA8 , NDUFA6 , SURF1 , ATP5F1A , NDUFB2 , MRPS30 , CYC1 , MRPL15 , DLAT , ATP5F1D , ACADSB , MRPS22 , SDHB , MRPL11 , PMPCA , AFG3L2 , MRPS11
**GLYCOLYSIS**	0.162442	0.246125	0.402943	1.175441	MIOX , KIF20A , DEPDC1 , CENPA , HMMR , PPFIA4 , COL5A1 , ME1 , B3GNT3 , STC2 , QSOX1 , STC1 , STMN1 , CAPN5 , CXCR4 , G6PD , HK2 , PGM2 , TPBG , SDC3 , SRD5A3 , EGLN3 , PKM , CD44 , DSC2 , FUT8 , PFKP , GPC1 , ELF3 , B4GALT4 , PLOD2 , AGRN , CACNA1H , PDK3 , ALDOA , B4GALT2 , KIF2A , CASP6
**REACTIVE OXYGEN SPECIES PATHWAY**	0.685786	0.75636	0.337832	0.862515	PRDX2 , PDLIM1 , G6PD , SRXN1 , GPX3 , PFKP , SOD2 , CDKN2D , ABCC1 , SBNO2
**P53 PATHWAY**	0.146817	0.236802	0.408319	1.190067	INHBB , ST14 , RGS16 , S100A4 , UPP1 , ACVR1B , GM2A , NUPR1 , ABCC5 , ITGB4 , GADD45A , RRAD , SFN , CDKN1A , LIF , GPX2 , RAP2B , IFI30 , CTSD , DRAM1 , ATF3 , TGFB1 , PTPN14 , APAF1 , CCND3 , PDGFA , RALGDS , BAK1 , RNF19B , LRMP , DGKA , TAX1BP3 , AEN , BAIAP2 , VAMP8 , BTG1 , HMOX1 , NDRG1 , PLK2 , ADA

**UV RESPONSE UP**	0.306069	0.413607	0.384858	1.101867	SLC6A8 , HSPA2 , EPCAM , CA2 , CCNE1 , PLCL1 , RRAD , NR4A1 , CHKA , PRKCD , ATF3 , ABCB1 , SQSTM1 , GPX3 , CCND3 , BAK1 , SOD2 , CTSV , AGO2 , ALDOA , BTG3 , BTG1 , AMD1 , HMOX1 , TYRO3 , TFRC , RFC4 , RASGRP1
**UV RESPONSE DN**	0.007805	0.017739	0.512606	1.462908	HAS2 , RGS4 , COL1A1 , SERPINE1 , PMP22 , CCN1 , RUNX1 , GJA1 , COL1A2 , MYC , IGF1R , GCNT1 , ATP2B4 , COL3A1 , FBLN5 , PDGFRB , FHL2 , COL5A2 , EFEMP1 , ITGB3 , MAP1B , SLC7A1 , KIT , LPAR1 , F3 , LAMC1 , ERBB2 , PIK3CD , ICA1 , CACNA1A , SMAD3 , CDON , ABCC1
**ANGIOGENESIS**	0.004279	0.01087	0.679476	1.64597	LPL , S100A4 , TNFRSF21 , TIMP1 , COL3A1 , STC1 , SPP1 , JAG1 , FGFR1 , COL5A2 , OLR1 , LUM , PDGFA , ITGAV , SLCO2A1
**HEME METABOLISM**	0.001129	0.005133	0.528329	1.539843	SPTA1 , KEL , SLC4A1 , GATA1 , XK , SLC6A8 , ADD2 , TRIM58 , ALAS2 , CA1 , SLC6A9 , SNCA , RHAG , HBQ1 , TRIM10 , TMCC2 , CA2 , PRDX2 , KLF1 , RBM38 , AGPAT4 , NFE2 , ACP5 , E2F2 , ARL2BP , UCP2
**COAGULATION**	0.27932	0.387944	0.394987	1.120281	THBS1 , SERPINE1 , ANXA1 , MMP11 , CAPN2 , PDGFB , CD9 , TIMP1 , TFPI2 , MMP2 , CAPN5 , GSN , PLAU , CTSH , VWF , SPARC , ITGB3 , OLR1 , F3 , CTSB , CLU , PLEK , CFD , CTSV , MMP15 , CSRP1 , COMP , ADAM9
**IL2 STAT5 SIGNALING**	0.000825	0.004124	0.541918	1.576013	GATA1 , ITIH5 , CDC6 , UCK2 , EMP1 , CAPN3 , RGS16 , ETV4 , CA2 , CCNE1 , CAPG , SYT11 , MYC , IGF1R , CD83 , TNFRSF21 , TLR7 , SLC1A5 , SPP1 , KLF6 , CKAP4 , GLIPR2 , PLIN2 , HK2 , CD79B , LIF , CD86 , CD44 , GPR65 , FGL2 , ABCB1 , TNFRSF1B , CISH , PHLDA1 , TRAF1 , CCND3 , PIM1 , APLP1 , IL10RA , SELP , ITGAV , BCL2L1 , ODC1 , CDCP1 , IGF2R , ENPP1 , NDRG1 , SELL , SERPINB6 , ENO3 , MYO1E , ADAM19 , ITGAE , TNFRSF4 , MAP3K8
**BILE ACID METABOLISM**	0.00146	0.005615	-0.55295	-2.21076	DHCR24 , SERPINA6 , AKR1D1 , TTR , HSD3B7 , PECR , CYP8B1 , PHYH , ALDH1A1 , NUDT12 , IDI1 , CROT , ALDH8A1 , HSD17B4 , AMACR , HSD17B6 , SOD1 , SLC27A2 , CAT , ABCA2 , PEX11G , GNMT , HSD17B11 , PRDX5 , SLC27A5 , ALDH9A1 , ACSL1 , GC , RXRA , ABCD1 , IDH2 , HAO1 , ABCA6 , LIPE , AGXT , LONP2 , HACL1 , GSTK1 , PEX16 , APOA1 , SLC23A1 , PEX11A , NR3C2 , IDH1 , ABCG8 , ISOC1 , BBOX1 , NR1I2 , PEX19
**PEROXISOME**	0.011568	0.023136	-0.38524	-1.50922	DHCR24 , SERPINA6 , TTR , HSD3B7 , ALDH1A1 , IDI1 , HSD17B4 , ACOX1 , SOD1 , SLC27A2 , CAT , ECI2 , ALB , HSD17B11 , PRDX5 , ALDH9A1 , ACSL1 , ACAA1 , ABCD1 , IDH2 , LONP2 , HMGCL , GSTK1 , CRABP1 , FDPS , SLC25A4 , PEX11A , DHRS3 , IDH1 , ISOC1 , CRAT , NR1I2
**ALLOGRAFT REJECTION**	0.000208	0.001299	0.577446	1.666237	CCL7 , CCL2 , ACHE , CCL22 , INHBB , CCL4 , IGSF6 , CAPG , CCR2 , CCR5 , TIMP1 , GCNT1 , IL18 , LY75 , TLR2 , ELF4 , MAP4K1 , LIF , TNF , CD86 , PRKCB , ITK , CD79A , CCR1 , GPR65 , IFNGR2 , SPI1 , CD8A , NLRP3 , STAT4 , TGFB1 , APBB1 , CCND3 , HCLS1 , ST8SIA4 , CTSS , ITGAL , FLNA , BCL3 , WAS , SRGN , IL12RB1 , TGFB2 , IRF7 , AKT1 , TLR6 , PTPRC , FYB1 , EIF4G3 , LCP2 , ITGB2 , NCF4 , PRKCG , IRF4 , CD74
**SPERMATOGENESIS**	0.000336	0.001866	0.636758	1.731628	KIF2C , DDX4 , CCNB2 , BUB1 , NPY5R , TNNI3 , HSPA2 , CFTR , ACE , NEK2 , PACRG , NCAPH , CDKN3 , PCSK4
**KRAS SIGNALING UP**	0.000207	0.001299	0.56508	1.636581	ADAMDEC1 , ADAM8 , HKDC1 , CCL20 , EMP1 , MMP11 , TMEM100 , GPNMB , CPE , RGS16 , GPRC5B , ETV4 , CA2 , EPB41L3 , ACE , MMD , APOD , GFPT2 , IL1RL2 , SPP1 , SPARCL1 , KIF5C , CXCR4 , F2RL1 , IL7R , PRDM1 , MAP4K1 , LIF , PLAU , LAPTM5 , LCP1 , TLR8 , DOCK2 , CMKLR1 , ABCB1 , BPGM , TNFRSF1B , TRAF1 , IKZF1 , DCBLD2 , CD37 , TNFAIP3 , BIRC3 , IL10RA , TRIB2 , FCER1G , CTSS , PLAUR , NIN , ETV5 , STRN , SPON1 , ANKH , MAP3K1
**KRAS SIGNALING DN**	0.383377	0.504444	0.386253	1.062264	EFHD1 , TNNI3 , SCN10A , HSD11B2 , SYNPO , BARD1 , SKIL , MFSD6 , MX1 , RSAD2 , SLC29A3 , ATP4A , EDN1 , CNTFR , MAST3 , TGFB2 , RYR1 , MYO15A , SPTBN2
**PANCREAS BETA CELLS**	0.695851	0.75636	0.36915	0.834259	ABCC8 , AKT3

Supplementary Table S4. Reactome “Fatty Acid Metabolism” gene set in each model. Genes included in the manuscript are highlighted (in yellow). Pval: p-value, Padj: p-adjusted, ES: enrichment score, NES: normalized enrichment score.

	Pval	Padj	ES	NES	Leading Edge
**Guinea pig**	0.003145	0.044609	-0.47078	-1.97238	THRSP, CYP1A2, ELOVL2, PTGDS, PECR, PON3, CYP8B1, HSD17B3, PHYH, PON1, CROT, ACOT12, HSD17B4, ACOX1, AMACR, ACSL3, CYP1A1, SLC27A2, DECR2, ECI2, ECHS1, CPT1A, DECR1, ACSL1, ECI1, HADH, CYP4B1, ACOT4, ACOT2, ACOT1, ACAA1, ALDH3A2, RXRA, ABCD1, ACADS, ACAD11, HPGD, SLC25A1, ACADM, HACL1, PTGR2, CYP4F11, CYP4A22, CYP4A11, ACOX2, ACAD10, MMAA, PCCA, PON2, SLC27A3, SLC25A20, ACBD5, MCEE, AKR1C3, PTGS1, CPT1B, CRAT, ACAA2
**HNASH 2**	0.003472	0.06584	-0.55709	-1.73678	CYP2C19, CYP1A2, THRSP, ACSM3, SCD, HAO2, HADH, ACADS, ACSL5, CYP1A1, RXRA, PECR, EPHX2, ECI1, CYP4F11, SLC25A1, HADHA, ACOX2, CYP8B1, HSD17B8, ECI2, ACSL1, PTGR1, MLYCD, ACAA2, HPGD, MCEE, MECR, SLC27A2, FASN, FADS2, ACOT13, MCAT, TECR, CRAT, ACACA, ACADL, PON3, ACSF2, CYP2C8, ALDH3A2, ACOT4, ECHS1, HACD3, ACOX1, NDUFAB1, CPT2, FAAH, CYP2C9, DECR1, ACAA1, CYP2J2, PCCB, NUDT19, AKR1C3, PON1, DECR2, HACL1, CPT1A, SLC25A20, CBR1, GPX4, AMACR, SLC25A17, ACBD4, GPX1, ELOVL5, PTGES2, ACACB, CYP4F3, DPEP2, ELOVL6, ACOT8
**HNASH 1**	0.858668	0.952725	0.194743	0.816251	ACSL4, PRKAA2, PTGDS, CYP4F22, GPX2, ELOVL2, THRSP, SCD, ACOT12, ACSL6, FASN, ACOT1, ELOVL6, FADS2, AKR1C3, DBI, PRXL2B
